# Dissecting Long-Term Glucose Metabolism Identifies New Susceptibility Period for Metabolic Dysfunction in Aged Mice

**DOI:** 10.1371/journal.pone.0140858

**Published:** 2015-11-05

**Authors:** Anuradha Chauhan, Heike Weiss, Franziska Koch, Saleh M. Ibrahim, Julio Vera, Olaf Wolkenhauer, Markus Tiedge

**Affiliations:** 1 Department of Systems Biology and Bioinformatics, Institute of Computer Science, University of Rostock, Rostock, Germany. Stellenbosch Institute for Advanced Study (STIAS), Wallenberg Research Centre at Stellenbosch University, Stellenbosch, South Africa; 2 Department of Medical Biochemistry and Molecular Biology, University of Rostock, Rostock, Germany; 3 Institute of Experimental Dermatology, University of Lübeck, Lübeck, Germany; University of Nebraska Medical Center, UNITED STATES

## Abstract

Metabolic disorders, like diabetes and obesity, are pathogenic outcomes of imbalance in glucose metabolism. Nutrient excess and mitochondrial imbalance are implicated in dysfunctional glucose metabolism with age. We used conplastic mouse strains with defined mitochondrial DNA (mtDNA) mutations on a common nuclear genomic background, and administered a high-fat diet up to 18 months of age. The conplastic mouse strain B6-mt^FVB^, with a mutation in the *mt-Atp8* gene, conferred β-cell dysfunction and impaired glucose tolerance after high-fat diet. To our surprise, despite of this functional deficit, blood glucose levels adapted to perturbations with age. Blood glucose levels were particularly sensitive to perturbations at the early age of 3 to 6 months. Overall the dynamics consisted of a peak between 3–6 months followed by adaptation by 12 months of age. With the help of mathematical modeling we delineate how body weight, insulin and leptin regulate this non-linear blood glucose dynamics. The model predicted a second rise in glucose between 15 and 21 months, which could be experimentally confirmed as a secondary peak. We therefore hypothesize that these two peaks correspond to two sensitive periods of life, where perturbations to the basal metabolism can mark the system for vulnerability to pathologies at later age. Further mathematical modeling may perspectively allow the design of targeted periods for therapeutic interventions and could predict effects on weight loss and insulin levels under conditions of pre-diabetic obesity.

## Introduction

**Fig 1:**
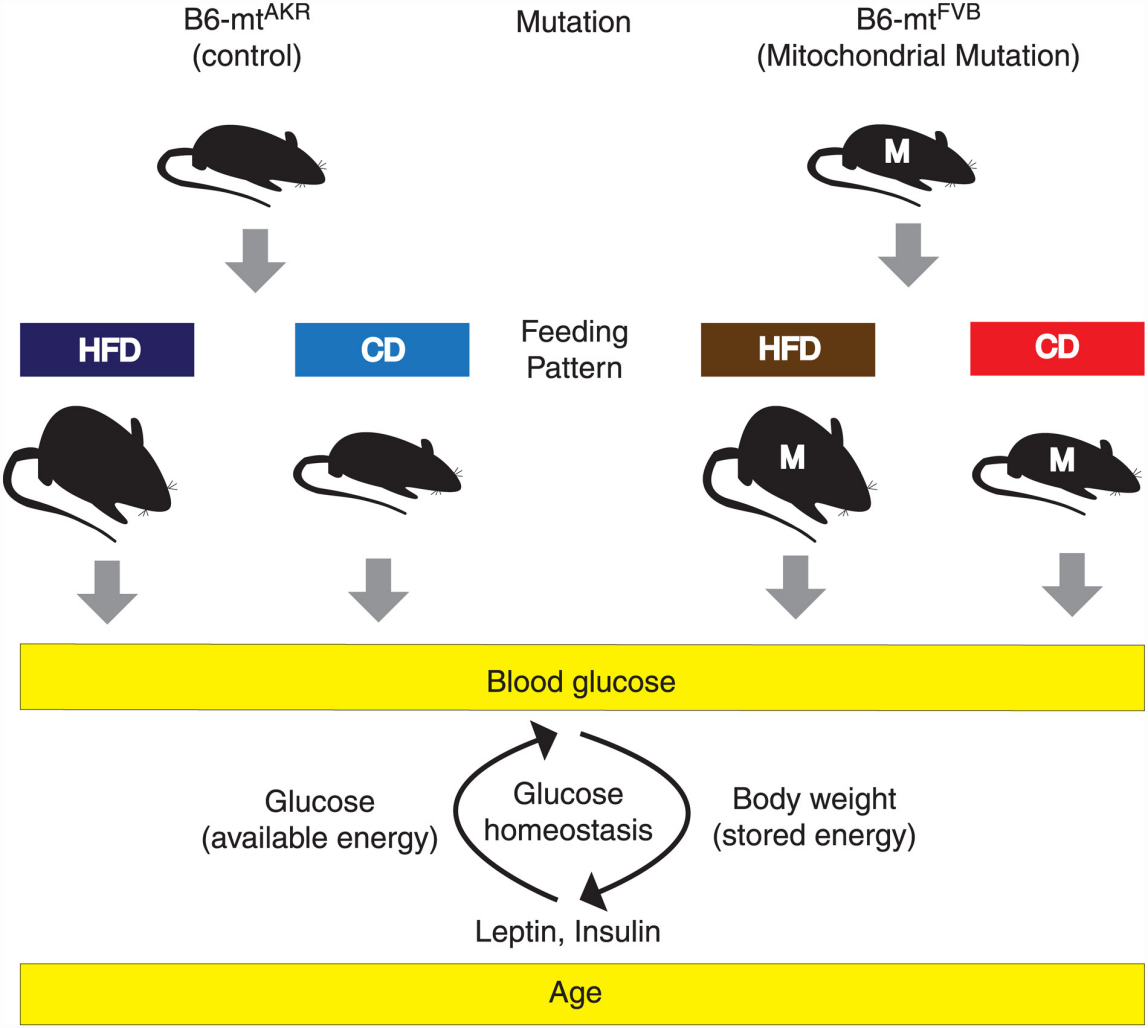
Experimental setup. The conplastic mouse strains B6-mt^FVB^ (*Atp8* mutation) and B6-mt^AKR^ (control) received high-fat diet (60% calories from fat) (HFD) or control diet (CD) (10% calories from fat) for up to 18 months months after weaning. Body weight and blood glucose levels were measured monthly. Serum leptin and serum insulin levels were monitored till 18 months in an interval of 3 months.

Metabolic disorders, like diabetes and obesity, are pathogenic outcomes of metabolic imbalance in glucose metabolism [1]. Imbalance in glucose metabolism can arise from a complex set of factors including genetic predisposition, nutrient excess and the ability of the body to deal with nutrient excess. The body’s ability to deal with excess nutrients involves direct adjustments in energy intake and expenditure pathways, along with the modulation in mitochondrial capacity/efficiency to generate ATP [2]. Mitochondria house their own genome (mtDNA), which can accumulate point mutations in an age-dependent manner in humans [3]. Conplastic mouse strains, with the nuclear genome of one strain and the mitochondrial genome of another, are valuable models to study the role of mitochondrial mutations [4,5]. To gain better insights into the impact of nutrient excess on the pathophysiology of disease with age, we administered a high-fat diet to conplastic mouse strains for 12 months. Conplastic mouse strains were generated by crossing mitochondrial genomes of common inbred strains on the popular B6 background. The conplastic mouse strain B6-mt^FVB^, has a stable mutation in *mt-Atp8* gene, found in mitochondrial ATPsynthase complex. A B6-mt^AKR^ strain was used as a control strain, which just differs for this Atp8 polymorphism from the B6-mt^FVB^ mtDNA sequence [6]. Fig 1 illustrates the experimental setup.

High-fat diet administration in the B6-mt^FVB^ strain, with compromised mitochondria, conferred β-cell dysfunction and impaired glucose tolerance in comparison to the B6-mt^AKR^ control strain [7]. Despite of these functional deficits, the blood glucose levels, unexpectedly, showed sensitivity to high-fat diet only during early age, followed by an adaptation. In order to understand this non-linear blood glucose dynamics, we further studied the effect of a 12 month long high-fat diet (HFD) administration on further key regulators of metabolism and subjected the data to mathematical modeling.

**Fig 2:**
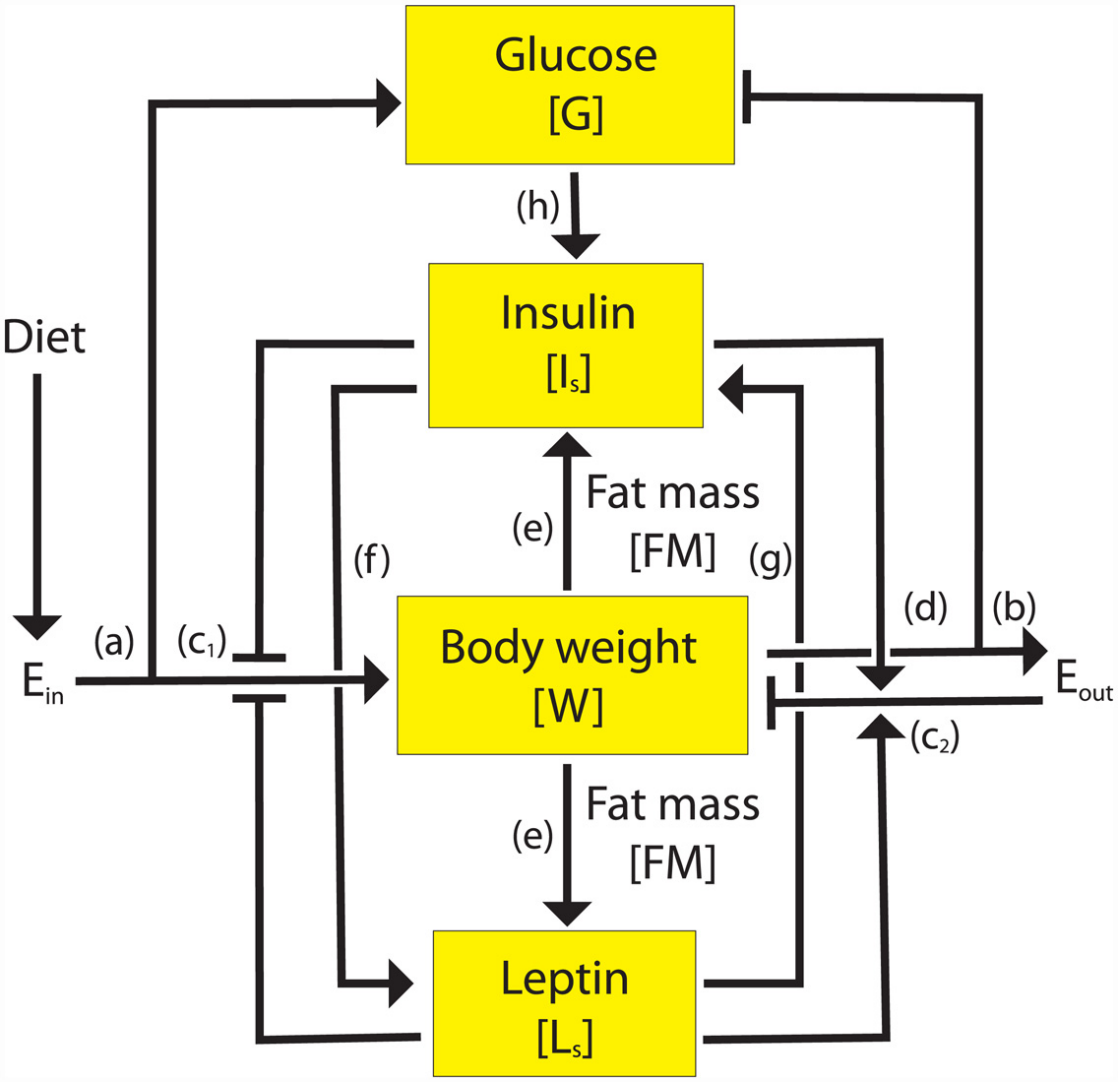
Model for diet-induced long-term glucose metabolism. Blood glucose levels (*G*) and body weight (*BW*) are regulated by overall energy balance of the body, which is defined by energy intake (*E*_*in*_) (a) and -expenditure (*E*_*out*_) (b). *E*_*in*_ depends on diet and demand for food intake (*FI*) (c_1_). *FI* is negatively regulated by elevated insulin (*I*_*s*_) and leptin (*L*_*s*_) levels. *E*_*out*_ is determined by *BW* (d) and increased energy expenditure, via *I*_*s*_ and *L*_*s*_, forming a delayed-negative-feedback loop (c_2_). When *E*_*in*_ is in excess, *BW* and fat mass (*FM*) increase. Increased fat mass induces the secretion of both *I*_*s*_ and *L*_*s*_ (e). *I*_*s*_ levels are also linearly regulated by *G* (h). *L*_*s*_ and *I*_*s*_ also coregulate each other in a positive-feedback loop manner (f), (g).

Fig 2 shows the network of key regulators, operating at several organ levels, in controlling the glucose homeostasis. Glucose homeostasis induced by high-fat diet, involves various processes taking place at different organ levels, including the brain, liver, pancreas and adipose tissues. While liver and pancreas play a central role in glucose metabolism, the adipose tissue is specialized in storing glucose as fat [8]. Additionally, the brain, is able to observe excessive energy signals to modulate glucose and energy homeostasis [9]. Over nutrition leads to a metabolic state characterized by a strongly elevated energy intake, which exceeds energy expenditure. This situation results in accelerated fat mass buildup, which induces rise in leptin levels [10]. Leptin is circulating in blood in proportion to body fat mass [11]. The brain senses the excess fat mass through changes in leptin levels. The primary role of leptin is as negative feedback regulator of diet-induced energy levels. Accumulating evidences have established an analogous role of insulin in diet-induced energy homeostasis [10]. Also, insulin and leptin have been found to share several intracellular and neuronal signaling pathways [12] Based on this knowledge, we model insulin and leptin as redundant pathways, which signal brain to suppress hunger in response to increased fat, while simultaneously increasing energy expenditure by other tissues. Serum insulin is induced by increase in fat mass as well as directly regulated by blood glucose levels [13]. Additionally, we assume a positive feedback between insulin and leptin, based on the studies that insulin positively regulates the leptin levels [14,15,16,17] and that leptin positively regulates insulin levels [18,16,19] (Fig 2).

Several models dealing with glucose homeostasis at the molecular level have been discussed in reviews [20,21,22,23,8]. There are several theoretical models at the physiological level on the effect of diet on energy balance and body weight dynamics [24,25,26,27,28,29]. Few models of body weight dynamics are calibrated from experimental data [30,31] and clinical data [32]. There is also a theoretical model dissecting the oscillatory property of weight loss and regain during dieting [33]. Despite of glucose homeostasis being so crucial in regulating long-term changes like body weight dynamics and insulin dynamics at the similar timescale, there are no extensive experimental studies focusing on long-term changes in glucose homeostasis within the scale of months available, nor are there models focusing on the mechanism underlying fat-induced glucose homeostasis. We here develop such a model of glucose homeostasis at the whole-body level, predicting the response to high-fat diet administration.

## Materials and Methods

### Conplastic mice

Conplastic C57BL/6N-mtFVB/NJ (B6-mt^FVB^) and C57BL/6N-mtAKR/J (B6-mt^AKR^) were generated as described previously [4,6]. All mouse strains were housed at the central animal care facility of the Medical Faculty, University of Rostock, receiving conventional rodent chow (SSNIFF, Soest, Germany) and water *ad libitum*. The colonies were regularly monitored for murine pathogens according to The Federation of European Laboratory Animal Science Associations (FELASA) recommendations. All procedures of this study were performed according to the guidelines for the care and use of laboratory animals, and were approved and supervised by the Landesamt für Landwirtschaft, Lebensmittelsicherheit und Fischerei Mecklenburg-Vorpommern (LALLF) (Approval No. 7221.3–1.1-059/12).

### High-fat diet feeding

Metabolic stress was induced feeding high-fat diet (HFD) (60% of the kcal as fat, Altromin, Lage, Germany) or control diet (10% of the kcal as fat, Altromin, Lage, Germany) for up to 18 months starting after weaning [34]. For HFD protein content was 21.3%, carbohydrate content was: monosaccharide 8.7%, disaccharides 18.94%, and polysaccharides 6.23%. Crude fat was 34% and metabolic energy content was 5017.105 kcal/kg. For control diet protein content was 21.1% and carbohydrate content was: monosaccharaides 2.6%, disaccharides 13% and polysaccharides 14.3%. Crude fat was 4% and metabolic energy content was 3493.98 kcal/kg. The animals were kept in cohorts of 4 animals/cage and had access to food and water *ad libitum*. 16–20 animals per strain and diet were continuously monitored for body weight and blood glucose. 8–11 animals per strain and diet were used for the leptin and insulin measurement.

### Measurements

Blood glucose concentrations and body weight were examined every second week and monthly. Blood glucose was determined by glucose oxidase method (Freestyle™, Abbott, Wiesbaden, Germany). Serum samples were taken from 4–6 hours fasted animals for analysis of insulin and leptin concentrations at the age of 3–18 months. Serum insulin levels were quantified using an ultra sensitive mouse insulin ELISA kit following the manufacturer´s instructions (Mercodia, Uppsala, Sweden).

Leptin ELISA Serum leptin levels were quantified using the Quantikine Mouse Leptin ELISA kit following the manufacturer´s guidelines (R&D Systems, Abingdon, UK).

### Statistical analyses

All quantitative data are presented as mean values ±SEM (standard error of the mean). Comparisons between multiple groups were analyzed using one-way ANOVA and Sidak post-test. Comparisons between two different groups were analyzed using Student´s t-test. If p<0.05 the differences were considered statistically significant. All calculations were done using the GraphPad Prism program (GraphPad Inc., San Diego, CA).

### Mathematical model

A phenomenological model was generated (Fig 2) using the whole-body data at the physiological level (Fig 1). The phenomenological modeling approach allows merging of various processes taking place at different organ levels, including brain, liver, pancreas and adipose tissues. The model is based on overall energy balance of the body, which is regulated by neurocentric sensing of fat-mass accumulation via insulin and leptin.

Major model assumptions:

- The model only considers time-scales of weeks and months to monitor the metabolic changes related to weight gain during ageing. Events that occur on shorter time scales like food intake, absorption and corresponding changes in glucose levels, insulin levels, leptin levels, pulsatile insulin release from pancreas and gene expression changes are assumed to be instantaneous and continuous as demonstrated by other models at the similar level of abstraction [35].
- Mode of induction of insulin is similar to fat-mass-dependent mode of induction of leptin, based on several findings suggesting insulin to be an adiposity signal, reviewed in [12]. Also, mode of action of insulin in the brain and in other peripheral tissues regulating energy intake and energy expenditure is assumed to be similar to mode of action of leptin for the sake of simplicity.
- Peripheral anabolic effect of insulin is ignored, since the concerning data did not add any explanatory power to the adapting blood-glucose dynamics.
- Clearance of insulin by liver and periphery is assumed to be linear for the sake of simplicity.
- Energy spent in physical activity is negligible, since animals are kept under constant environmental condition without any physical challenge.
- Since serum leptin and insulin concentrations are typically much higher than leptin and insulin concentrations in the brain, loss of leptin and insulin, which is taken up into the brain, is not accounted for in the leptin and insulin levels in the serum.
- Any increase in β-cell size is ignored, such that increased β-cell mass only corresponds to increased β-cell numbers. Thus, an increase in β-cell mass is proportional to increase in β-cell numbers via proliferation. We assume a logistic increase of β-cell population, since logistic models are biologically reasonable in representing population growth [36].
- Low-grade inflammation is marked by an increase in adipose tissue macrophage population. We assume that the increase in macrophage population is proportional to changes in IL-6 levels, based on the evidence that, adipose tissue macrophages participate in pro-inflammatory pathways accounting for significant amount of IL-6 expression [37]. We model the dynamics of macrophage population by assuming logistic increase in the population.

The changes in blood glucose levels in the time scale of body weight change can be determined from changes in overall energy balance. The model is composed of the following four differential equations:

### Model Equations

$$\frac{d\left[G\right]}{dt}=\sigma_{diet}\;\cdot k_{ins}\cdot \left(\left[E_{in}\right]-\left[E_{out}\right]\right)-k_{16}\cdot \left[G\right]$$ (1)

$$\frac{d\left[FM\right]}{dt}=\sigma_{diet}\cdot \frac{\left[E_{in}\right]-\left[E_{out}\right]}{\rho_{fat}}$$ (2)

$$\frac{d\left[L_{s}\right]}{dt}=k_{8}\cdot \left[FM\right]+k_{9}\cdot \left[I_{s}\right]-k_{10}\cdot \left[L_{s}\right]$$ (3)

$$\frac{d\left[I_{s}\right]}{dt}=k_{11}\cdot {\left[G\right]}^{2}+k_{12}\left[FM\right]+k_{13}\cdot \left[L_{s}\right]-k_{14}\cdot \left[I_{s}\right]$$ (4)

where,

$$\left[E_{in}\right]=\sigma_{food}\cdot \left[FI\right]$$

$$\left[FI\right]=k_{4}\cdot \left(\left(1-\frac{\left[L_{brain}\right]}{k_{5ml}+\left[L_{brain}\right]}\right)+\left(1-\frac{\left[I_{brain}\right]}{k_{5mi}+\left[I_{brain}\right]}\right)\right)$$

$$\left[E_{out}\right]=k_{6}\cdot \left[BW\right]\cdot \left(1+\frac{k_{7l}\cdot \left[L_{brain}\right]}{k_{7m}+\left[L_{brain}\right]}+\frac{k_{7i}\cdot \left[I_{brain}\right]}{k_{7m}+\left[I_{brain}\right]}\right)$$

$$\left[L_{brain}\right]=k_{1}\cdot \frac{L_{s}}{k_{2}+L_{s}}+k_{3}\cdot L_{s}$$

$$\left[I_{brain}\right]=k_{1}\cdot \frac{I_{s}}{k_{2}+I_{s}}+k_{3}\cdot I_{s}$$

FI is food intake. *σ*_*diet*_ scales the satiety coefficient based on the type of diet administered (CD or HFD), where, *k*_*ins*_ is the parameter for fat mass-independent regulation of glucose by insulin, *σ*_*fat*_ is the energy density of fat, *σ*_*food*_ is the metabolizable energy content of food and *k*_*4*_ is the parameter for maximum food intake value. Values for each parameter used in this model are listed in Table 1. Detailed derivations of model equations and parameters are available in Supplemental Data (S1 File).

**Table 1:** Model parameters.

Parameter	Value	Source
*k*_*1*_	1.42	[35]
*k*_*2*_	15.6	[35]
*k*_*3*_	0.00272	[35]
*k*_*4*_	0.275	Fitted
*k*_*5ml*_	1.1	Fitted
*k*_*5mi*_	1.1	Fitted
*k*_*6*_	0.0018	Fitted
*k*_*7l*_	0.6	Fitted
*k*_*7i*_	0.4	Fitted
*k*_*7m*_	0.22	Fitted
*σ*_*diet*_ for CD	18.72	Fitted
*σ*_*diet*_ for HFD	18.72*1.4	Fitted
*σ*_*fat*_	9	[43]
*k*_*ins*_	0.778	Fitted
*k*_*8*_	0.195	Fitted
*k*_*9*_	0.02	Fitted
*k*_*10*_	0.213	Fitted
*k*_*11*_ for B6-mt^AKR^	0.132	Fitted
*k*_*11*_ for B6-mt^FVB^	0.132*0.1	Fitted
*k*_*12*_	0.0001	Fitted
*k*_*13*_	0.0011	Fitted
*k*_*14*_	0.066	Fitted
*k*_*15*_	0.045	Fitted
*r*_*hf*_	10	Fitted
*r*_*cd*_	0.1	Fitted
a	0.31	Fitted

## Results

### Blood glucose response and its key regulators

**Fig 3:**
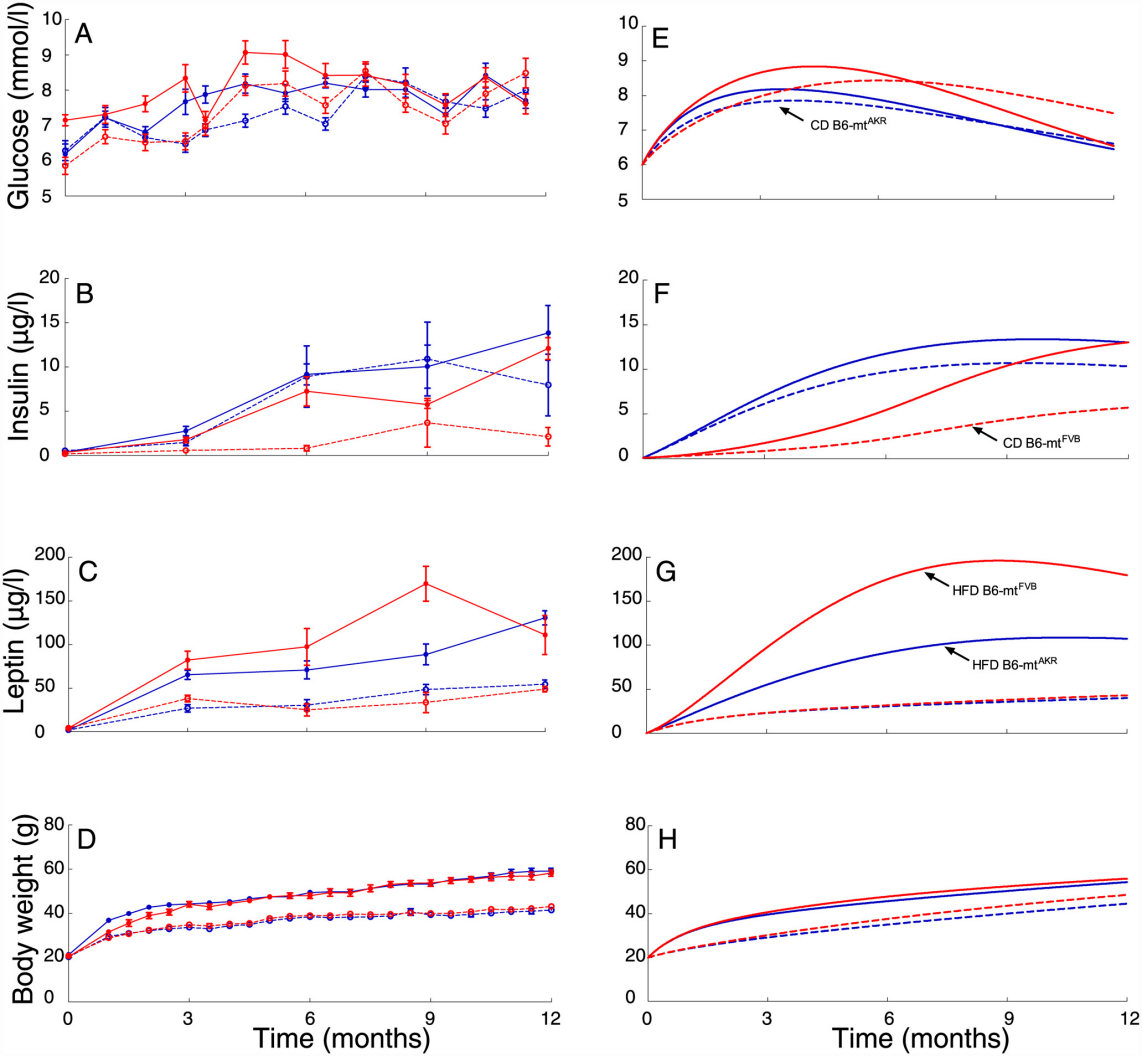
Time-courses of blood glucose, insulin, leptin and body weight in vivo and simulated. Blood glucose levels exhibited a characteristic two-peak response irrespective of diet and strain. There was a significant effect of high-fat diet (solid lines) on blood glucose response at early time points (3, 6 months of diet) in both control (B6-mt^AKR^ in blue) and mutated (B6-mt^FVB^ in red) strain, however with age the blood glucose levels adapted in all cases (A). Serum insulin levels were significantly lower in mutated strain under control diet (B6-mt^AKR^, red dashed line), while high-fat diet administration compensated for this lack of insulin by 6 months of age, which then remained elevated over 12 months of diet (red solid line) (B). Serum leptin levels were also significantly higher for mice fed high-fat diet, which further remained elevated over 12 months of diet (solid lines) (C). Mice fed with a high-fat diet had a pronounced increase in body weight (solid lines) throughout the 12 months of feeding compared to mice fed control diet (dashed lines) (D). Model simulations of respective blood glucose levels (E), serum insulin levels (F), serum leptin levels (G) and body weight (H). Shown are means + SEM from n = 8–12 mice per strain and diet.

Blood glucose levels responded strongly to HFD administration in the early-age till 6 months, followed by adaptation, in both B6-mt^FVB^ and B6-mt^AKR^ strains (Fig 3A). Serum insulin levels were drastically reduced in B6-mt^FVB^ strains under control diet (CD), suggesting impairment of insulin secretion due to mutation in the *mt-Atp8* gene, which encodes a protein exhibiting ATPase activity. High-fat diet administration to B6-mt^FVB^ mice, induced compensatory increase in insulin secretion (Fig 3B), by increasing the proliferation of insulin secreting β-cells, as suggested by our previous work [7]. Leptin levels in HFD fed B6-mt^FVB^ and B6-mt^AKR^ strains had a considerably earlier onset and sustained higher levels in the late age (Fig 3C). Body weight increased with age in HFD fed mice over 12 months (Fig 3D). Model simulations were in agreement with strong effects of HFD during the early period of diet administration (Fig 3E). Leptin and insulin simulations were also in accordance with experiments and showed considerably elevated levels in the late age under high-fat diet scenario (Fig 3F and 3G). Simulation of body weight also agreed with significantly increasing body weight for high-fat diet in comparison to control diet (Fig 3H). In the following section we elaborate how mathematical modeling can help in explaining the essential role of fat-induced insulin and leptin, determining the observed early age strong response to HFD, and revealing a second peak in glucose levels between 15 and 21 months.

### Insulin and leptin have strong regulatory effects on the blood glucose response

**Fig 4:**
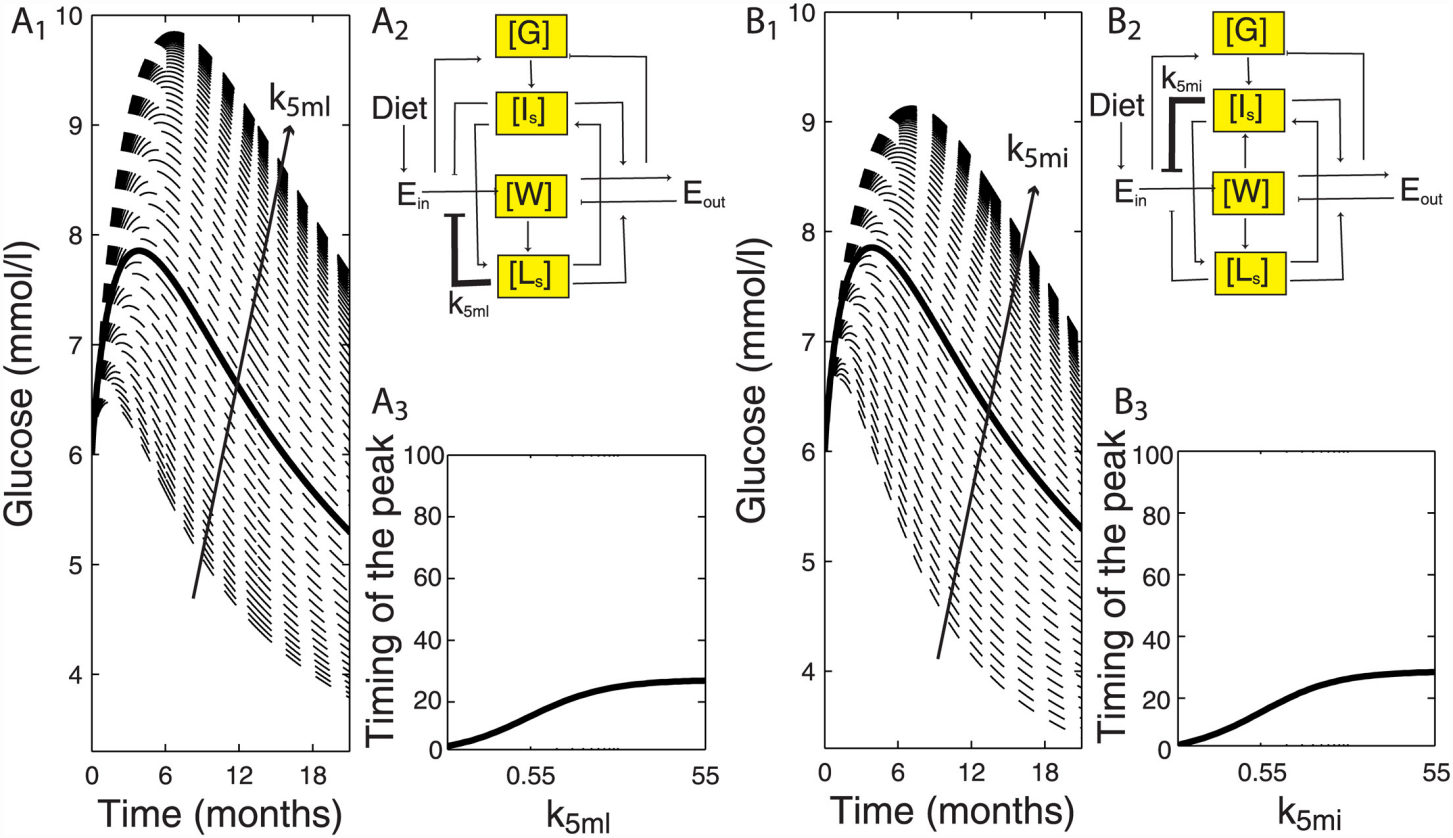
Insulin and leptin strongly regulate the timing of blood glucose peak. Leptin (*k*_*5ml*_) and insulin (*k*_*5mi*_) mediated control of demand for food-intake via E_in_ (A_2_, B_2_). Blood glucose response to 100-fold change in *k*_*5ml*_ and *k*_*5mi*_ (dashed lines) compared to default blood glucose response (bold line). Arrow depicts the direction of fold-change increase in *k*_*5ml*_ and *k*_*5mi*_ (A_1_, *B*_*1*,_). Timing of the blood glucose peak is equally sensitive to fold change in respective leptin and insulin parameters that determine demand for food intake (A_3_, B_3_).

**Fig 5:**
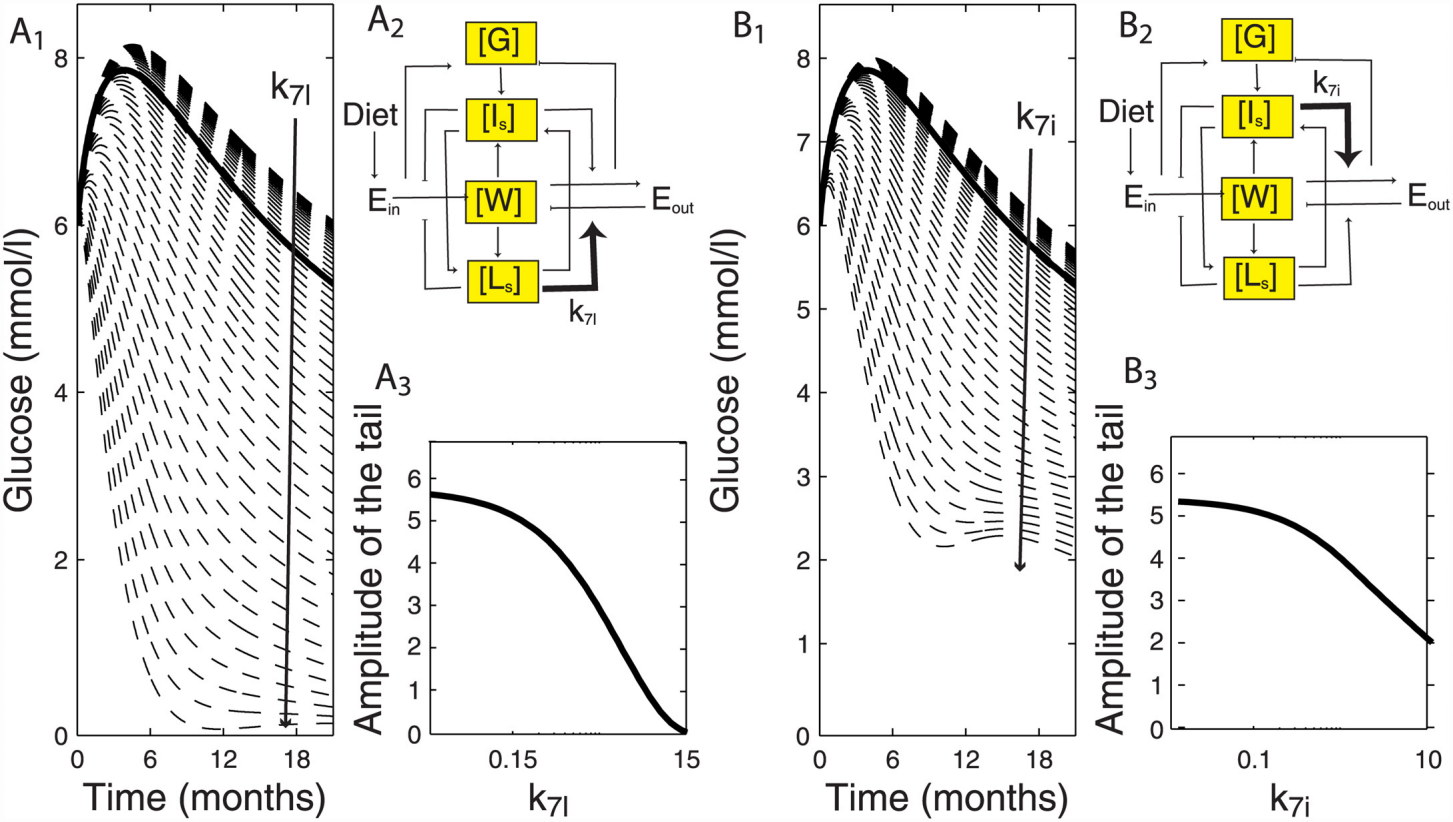
Leptin- and insulin-mediated negative-feedbacks control the blood glucose response. Insulin (*k*_*7i*_) and leptin (*k*_*7l*_) mediated negative-feedback regulating energy expenditure via E_out_ (A_2_, B_2_). At the later age, leptin has a stronger impact on the blood glucose dynamics. Arrow depicts the direction of fold-change increase in *k*_*7i*_ and *k*_*7l*_ (A_1_, *B*_*1*,_). Amplitude of the blood glucose tail is more strongly affected by leptin-mediated negative-feedback (*k*_*7l*_) compared to insulin-mediated negative-feedback (*k*_*7i*_) (B_3_, A_3_). Sufficiently strong negative-feedback (e.g. 50 fold increase in *k*_*7l*_, and *k*_*7i*_) leads to a second rise in blood glucose levels (A_1_ and B_1_).

In order to delineate the regulatory effects of insulin, leptin and body weight on the blood glucose dynamics, we performed a sensitivity analysis by systematically varying the model parameters over a 100-fold range (Figure C in S1 File). Both leptin (Fig 4A_1_) and insulin (Fig 4B_1_) were equally crucial in determining the overall blood glucose dynamics, when demand for food intake (k_5ml_, k_5mi_) (Fig 4A_2_ and 4B_2_) was varied. Timing of the early age blood glucose peak was strongly controlled by both leptin and insulin (Fig 4A_3_ and 4B_3_). However, leptin had a stronger impact on blood glucose dynamics at a later age (Fig 5A_1_), compared to insulin (Fig 5B_1_), when energy expenditure via leptin and insulin (k_7l_, k_7i_) (Fig 5A_2_ and 5B_2_) was varied in the same range. Amplitude of the blood glucose tail decreased more for leptin mediated energy expenditure with the fold change in respective parameter (Fig 5A_3_), compared to insulin mediated energy expenditure (Fig 5B_3_). Note that the simulations are performed for longer than 12 months of age.

### Adaptation of blood glucose response with age

**Fig 6:**
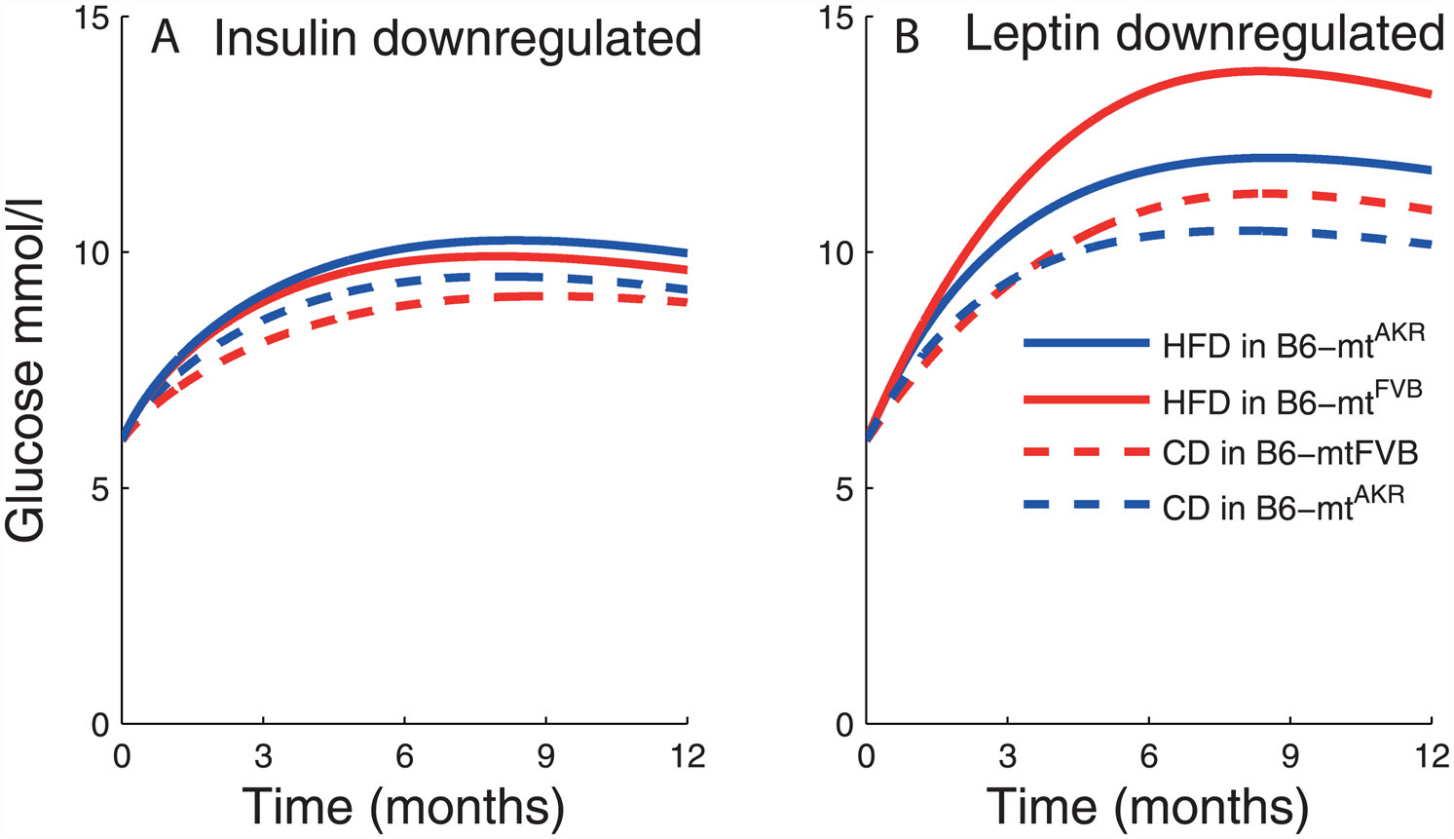
Redundancy of insulin and leptin pathways allows more effective adaptation of blood glucose response with age. Redundancy of insulin pathway and leptin pathway was removed by alternately downregulating the insulin and leptin derivatives to zero. The blood glucose levels remained elevated at the later age, indicating compromised adaptation, in all cases for both insulin downregulation (A) and leptin downregulation (B).

The conplastic mouse strain B6-mt^FVB^, carrying the mt-Atp8 mutation, confers β-cell dysfunction and impaired glucose tolerance after high-fat diet [7]. Despite such a functional deficit, the glucose homeostasis to our surprise, started to adapt with age irrespective of strain and diet (Fig 3). Mechanistic insights gained through the sensitivity analysis of the model suggest that leptin and insulin together are important regulators not only in the determining the early age peak of blood glucose levels but also influencing their later age dynamics (Figs 4 and 5). We further asked how crosstalk between insulin and leptin plays a role towards adaptation of blood glucose dynamics in the later age. We broke the redundancy of insulin and leptin pathways in the model by downregulating them alternatively, such that either insulin predominates or leptin predominates the regulation at a time. Insulin and leptin downregulation was simulated by putting their respective derivatives to zero. The blood glucose levels remained significantly elevated at 12 months, compared to the default time-course (Fig 3A), for both insulin downregulation (Fig 6A) and leptin downregulation (Fig 6B), implicating that the later age adaptation of blood glucose levels is compromised (Fig 6). Thus, modeling results suggest that redundancy of insulin and leptin pathways serves a survival advantage by ensuring that the blood glucose levels adapt better with age to challenges like high-fat diet administration and mitochondrial mt-Atp8 mutation.

### Two sensitive periods in long-term glucose homeostasis

**Fig 7:**
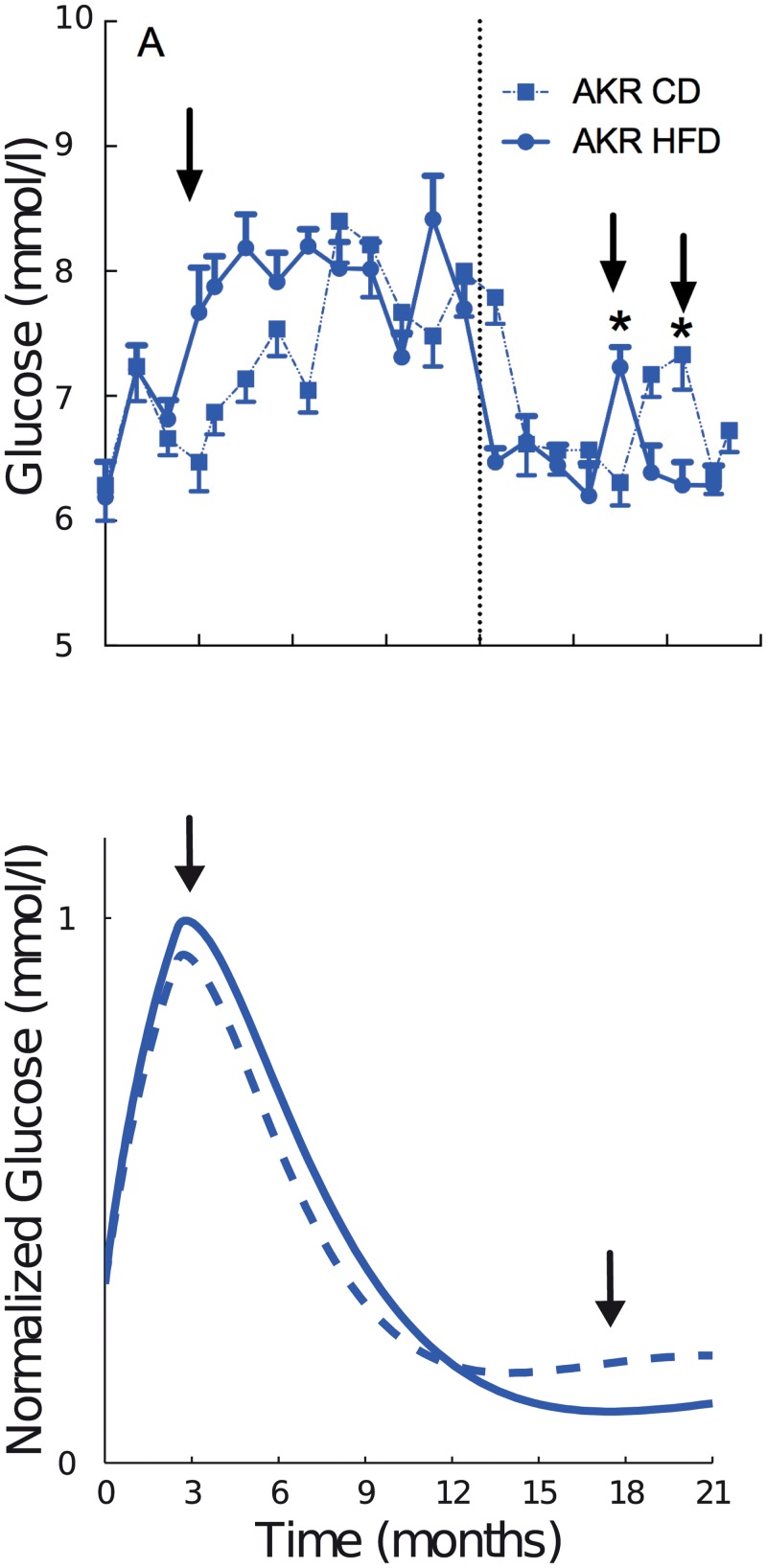
Two-peak blood glucose response. Prolonged feeding of HFD and CD till 18 months of age confirmed the second minor peak in blood glucose levels between 15 to 21 months (A), which was predicted by the second minor rise in the blood glucose simulations after 15 months. Simulations were performed by 50-fold increasing the insulin- and leptin-mediated delayed-negative-feedback (k_7l_, k_7i_) (B). Note that the simulations depict normalized glucose values. Shown are means ±SEM from n = 8–12 mice per diet. *p < 0.05 ANOVA plus Sidak’s post test comparing blood glucose levels from month 12–18.5 months (A).

Simulations in Fig 5 suggest a second rise in blood glucose levels after 12 months of age when both the leptin- and insulin-mediated delayed-negative-feedback parameters (k_7l_, k_7i_) are increased 50-fold (Fig 5A_1_ and 5B_1_). We performed further experiments till later time points of 18 months and indeed observed a second minor peak of glucose at the later age between 15 and 21 months for both control as well as high-fat diet scenario (Fig 7A). This peak around 16.5 month could be statistically verified from experimental data (n = 8–12, p < 0.05 ANOVA plus Sidak post test, comparing blood glucose levels between 12 and 18 months) (Fig 7A).

We asked whether this second minor peak could be explained by the feedback loop architecture of the model. Increase in energy expenditure comprises a negative feedback on fat-mass accumulation, and can only be activated after sufficient levels of respective insulin and leptin are present. This causes a delay in activating the negative feedback. Delayed-negative feedbacks are the regulatory motifs, which are known to induce oscillations [38]. Oscillations in glucose metabolism are well known at different timescales, like, pulsatile, dietary, ultradian. The model topology involving a delayed negative feedback, suggested that secondary peak in blood glucose levels could be an inherent oscillatory property at a longer timescale of body-weight induced dynamics during aging. When the strength of both leptin and insulin mediated negative feedback (k_7l_, k_7i_) was increased (20 times for CD and 50 times for HFD), the model simulations showed a second minor rise in blood glucose levels under control as well as high-fat diet scenario (Fig 7B). However, at its present level of abstraction, the model does not represent the timing and amplitude of the peaks exactly, but qualitatively explains the characteristic two peak dynamics of the blood glucose response. In case of B6-mt^FVB^, insulin levels are greatly influenced by increased β-cell mass proliferation. Since our focus is on body weight-induced insulin and leptin, we do not discuss the B6-mt^FVB^ strain for secondary peak in this work. Taken together, the modeling insights suggested a two-peak blood glucose response, which was confirmed by the experiments. The model explains this response to be an inherent oscillatory character of the system, enabled by insulin- and leptin mediated delayed-negative-feedback loop.

## Discussion

An early age responsiveness of blood glucose levels to experimental perturbations followed by an unexpected adaptation at the later age, had motivated us to perform mathematical modeling to delineate the regulatory effects of insulin and leptin. Modeling provided insights into how feedback regulations and cross talk among these pathways could shape the non-linear blood glucose response. Model predictions led to further experiments, which confirmed the occurrence of a second minor peak, where again insulin- and leptin-mediated negative feedback was important. Insulin and leptin are the signals that directly restore the energy balance through long-term glucose homeostasis. Both have been successfully used in therapeutic research for diabetes and obesity for decades [39,40]. Dissecting the regulation of long-term glucose homeostasis via pathways involving both insulin and leptin would be critical to understanding progression of metabolic diseases. Only then can an effective therapeutic intervention be designed. So far, both insulin and leptin therapies have their limitations, especially when it comes to long-term administration [41,42]. However, there is a long way before therapeutic interventions using both insulin and leptin can be designed. Meanwhile, knowledge on combined leptin- and insulin-mediated regulation of glucose metabolism and body weight should be refined through focused experiments. Easy monitoring and administration of both insulin and leptin already reduces some experimental burden. Furthermore, modeling methodologies should be improved to handle data in such a way that parameter values can be estimated directly from experimental data to ensure a better physiological interpretation.

The blood glucose response is sensitive to perturbation only in early age followed by an adaptation. Despite this adaptation, high-fat diet administration in B6-mt^FVB^ strain conferred β-cell dysfunction and impaired glucose tolerance at the later age [7]. Mitochondrial dysfunction induced by mutation of the Atp8 protein within the ATP synthase resulted in a reduced glucose responsiveness of insulin secretion and impaired glucose tolererance [7]. Concomitantly, metabolic stress resulted in an adaptive increase of beta cell mass. B6-mt^FVB^ mice were prone to development of hepatosteatosis, continuous glucose intolerance ultimately shortening the life span of the animals (F.K. manuscript in preparation). Interestingly, all mouse strains showed insulin resistance after feeding a high fat diet. Thus, impaired insulin action appeared not to be predictive for development of hepatosteatosis, beta cell mass regulation at the age > 6 months and ultimately also life span under conditions of obesity (F.K. manuscript in preparation). Overall our data and models support the accumulating evidence that metabolic changes during early age can have marked modulating effects on health in later life. This concept termed as metabolic programming, introduces limited, sensitive periods of early development that could provide the initial trigger for increased susceptibility to the range of adverse symptoms that we associate with the metabolic syndrome [24]. Our experiments with extended life period show two periods of sensitivity. One at the early age, complying with the known early age sensitivity period, second one at a much later age of 15–18 months, which has not yet been reported so far. A better understanding about regulation of the two sensitive periods of life through insulin and leptin, could in future, support a better planning for a programmed therapeutic intervention towards healthy living. Despite of their limitations, such physiological models could be an important first step, because diseases are manifested and diagnosed at the physiological level where the whole body is involved.

## Conclusion

The current study was conducted within the context of a much broader research to gain insights into the dynamics of age related metabolic diseases like obesity and diabetes. We presented a whole-body model of long-term glucose homeostasis at physiological level. Modeling provided insights into the insulin and leptin mediated regulation of two sensitive periods of glucose homeostasis. The sensitive periods, where experimental perturbations have maximum effects, can provide the initial trigger for increased susceptibility to metabolic diseases in the later age. Further experiments using programmed insulin and leptin administration and monitoring, combined with planned diet regimes, can improve our understanding about progression of metabolic diseases like obesity and diabetes. This could further help in designing therapeutic interventions to assess weight-loss and insulin control regimes.

## Supporting Information

S1 File Derivation of equations. IL-6 mRNA-expression at 3 and 12 months **(Figure A).** Increase in beta cell mass **(Figure B).** Sensitivity analysis **(Figure C).** Model parameters **(Table A).** Parameters for β cell mass proliferation **(Table B).** Parameters for high-fat diet induced low-grade inflammation **(Table C).** Initial conditions **(Table D).**
(DOCX)
