# Supplementary material for: Dissecting Long-Term Glucose Metabolism Identifies New Susceptibility Period for Metabolic Dysfunction in Aged Mice
Source: PLoS One. 2015 Nov 5;10(11):e0140858. doi: 10.1371/journal.pone.0140858 (PMC4634931; doi:10.1371/journal.pone.0140858)
Supplement: S1 File — IL-6 mRNA-expression at 3 and 12 months (Figure A). Increase in beta cell mass (Figure B). Sensitivity analysis (Figure C). Model parameters (Table A). Parameters for β cell mass proliferation (Table B). Parameters for high-fat diet induced low-grade inflammation (Table C). Initial conditions (Table D). (DOCX) [file pone.0140858.s001.docx]

**Supporting information**

**Derivation of equations**

Energy balance equations: Overall energy balance of the body is determined by energy intake (*E_in_*) and energy expenditure (*E_out_*). *E_in_* is dependent on the calories taken as food intake and demand for food intake. Demand for food-intake is produced by brain, based on the energy excess information brain receives [1]. Energy excess information is conveyed to the brain through hormones leptin and Insulin [2]. Increase in fat mass in the adipose tissue is a good indicator for energy excess. Thus, leptin and insulin deliver signals to the brain in an endocrine feedback loop that regulates body fat. Serum leptin enters the brain both by saturable receptors and by a non-saturable linear process [3]. Insulin also has receptors in the brain and there are evidences that it also uses similar processes like saturable transport to enter the brain [4]. Therefore, we assume similar mode of entry to brain for both insulin and leptin. Such mode of transport involving saturable and non-saturable transport can be represented by the following equations as per Tam et. al., 2009 [1].

The role of leptin in food intake is well known. The food intake is highest at low leptin levels, and decreases with rising leptin levels. Tam et. al., 2009 proposed a modified form of classic Michaellis-Menten equation to describe this relationship between leptin and food intake [1]. We assume a similar relationship between insulin and food intake, since insulin also regulates the food intake in a similar manner [2].

$$FI=k_{4}\cdot\left(\left(1-\frac{\left[L_{brain}\right]}{k_{5ml}+\left[L_{brain}\right]}\right)+\left(1-\frac{\left[I_{brain}\right]}{k_{5mi}+\left[I_{brain}\right]}\right)\right)$$

where, *FI* is food intake and *k_4_* is the parameter for maximum food intake value. Therefore, energy intake *E_in_* can be defined by the following equation

$$[E_{in}]=\sigma_{food}\cdot FI$$

where, *σ_food_* is the metabolizable energy content of food.

*E_out_* can be defined by energy spent in body weight-induced changes in metabolism and physical activity [3]. Energy spent in physical activity is assumed to be constant. The relationship between energy expenditure, body weight and body weight-induced changes in metabolism is less clear. However, it is known that insulin and leptin mediate in increasing utilization of energy in metabolic processes by several tissues. Based on several experimental observations, Tam et al. derived a relationship between energy expenditure, body weight and leptin [1]. We adapted this relationship to include a similar effect of insulin on energy expenditure. Without insulin and/or leptin energy expenditure is linearly proportional to body weight. Additional effect of leptin and insulin is modeled as a saturable function, such that energy-expenditure related effects of insulin and leptin are most prominent when their levels are low but become roughly constant at higher levels of leptin and insulin as per Tam et. al. 2009 [1].

$$[E_{out}]=k_{6}\cdot\left[BW\right]\cdot\left(1+\frac{k_{7l}\cdot\left[L_{brain}\right]}{k_{7m}+\left[L_{brain}\right]}+\frac{k_{7i}\cdot\left[I_{brain}\right]}{k_{7m}+\left[I_{brain}\right]}\right)$$

Body mass *(BW)* is a sum of fat mass *(FM)* and growth mass *(GM)*. *FM* represents the amount of energy stored as fat at time t, which can be defined by the overall energy balance.

$$\frac{d[FM]}{dt}=\sigma_{diet}\cdot\frac{[E_{in}]-[E_{out}]}{\rho_{fat}}$$

where, *σ_fat_* is the energy density of fat and *σ_diet_* scales the satiety coefficient, based on the type of diet administered (CD or HFD). *GM* is mass acquired due to physical growth of the body, which rises exponentially in the early age, followed by saturation in adulthood [4]. Based on this, we model the growth mass using first order delay equation.

Equations for serum leptin, serum insulin and blood glucose concentration [G]:

Serum leptin (*L_s_*): Leptin is produced and secreted by fat cells at a rate roughly linear to total fat tissue mass and cleared by the kidney in an insaturable manner through glomerular filteration [5]. Additionally, *I_s_* positively regulates the *L_s_* levels [6,7,8,9].

$\frac{d[L_{s}]}{dt}=k_{8}\cdot[FM]+k_{9}\cdot[I_{s}]-k_{10}\cdot[L_{s}]$

where, *k_8_* is the rate of leptin synthesis and k_10_ is the rate of clearance of leptin by kidney, *k_9_* is the strength of positive feedback via *I_s_*.

Serum insulin (*I_s_*): The major stimulant of insulin secretion is an increase of local glucose levels in the pancreas. Time scale of weeks and months allows us to model the glucose-induced insulin secretion to account only for the changes in glucose levels in response to body fat dynamics, based on the observations that the degree of glucose-stimulated insulin secretion is a direct function of body fat [2]. Additionally leptin positively regulates insulin levels [8,10,11], however the type of regulation is unclear. We therefore assume a linear regulation of insulin via leptin.

$$\frac{d[I_{s}]}{dt}=k_{11}\cdot[G]^{2}+k_{12}[FM]+k_{13}\cdot[L_{s}]-k_{14}\cdot[I_{s}]$$

Blood glucose (*G*): Changes in glucose levels on shorter timescale for daily food intake, absorption and pulsatile insulin release from pancreas are ignored. We only consider changes in blood glucose levels in the time scale of body weight change, which can be determined by changes in overall energy balance.

$$\frac{d[G]}{dt}=\sigma_{diet}\cdot k_{ins}\cdot([E_{in}]-[E_{out}])-k_{16}\cdot[G]$$

where, *σ_diet_* scales the satiety coefficient based on the type of diet administered (CD or HFD) and *k_ins_* is the parameter for fatmass-independent regulation of glucose by insulin.

High fat diet (HFD) stress: In order to simulate high-fat diet, satiety coefficient, *σ_diet_*, is scaled 1.4 times higher compared to control diet. High-fat-diet-induced obesity generates a state of low-grade inflammation, where adipose tissue macrophages infiltrate the adipose tissue [12,13,14]. These cells are a significant source of pro-inflammatory cytokines, like TNF-α and IL-6 (that can induce insulin resistance in adipocytes) [15]. It is well established that adipose tissue macrophages increase in numbers in diet-induced obesity, and participate in pro-inflammatory pathways accounting for significant amount of TNF-α and IL-6 expression [13]. We also observed a rise in IL-6 mRNA levels for both the strains under high-fat diet, indicating low-grade inflammation (Figure A in S1 file).


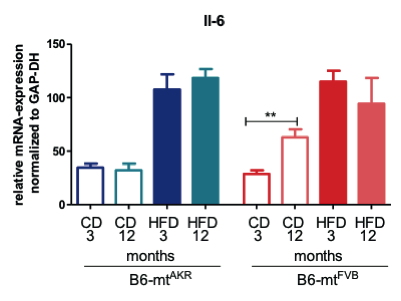


**Figure A.** **IL-6 mRNA-expression at 3 and 12 months.** mRNA expression for B6-mt^FVB^ strain (in red) and mRNA expression for B6-mt^AKR^ strain (in blue) under high-fat diet (filled boxes) and control diet (empty boxes).

We assume a logistic increase in adipose tissue macrophage population, since logistic models are biologically reasonable in representing population growth, like in case of tumors [16]. We assume that IL-6 levels represent the adipose tissue macrophage population dynamics.

$$\frac{d[Inflm]}{dt}=r_{i}\cdot\left(1-\frac{[Inflm]}{K_{i}}\right)\cdot[Inflm]$$

where, *r_i_* is the maximum growth rate of macrophage population and *K_i_*  scales the maximum population size that the adipose tissue can sustain.

Mitochondrial mutation (B6-mt^FVB^) stress: B6-mt^FVB^ is the mice strain with which is carrying a mutation in the ATP-synthase subunit. The mutation results in reduced glucose-induced insulin secretion due to dysfunctional ATP generation. We model this reduction in glucose-induced insulin secretion by reducing the respective parameter (*k_11_*). Also, reduced glucose-induced insulin secretion triggers an increase in β-cell mass as a compensatory mechanism. In order to cope with high-fat diet induced higher glucose levels, β-cell mass increase in B6-mt^FVB^ is more pronounced in high-fat diet fed mice (Figure B in S1 File).


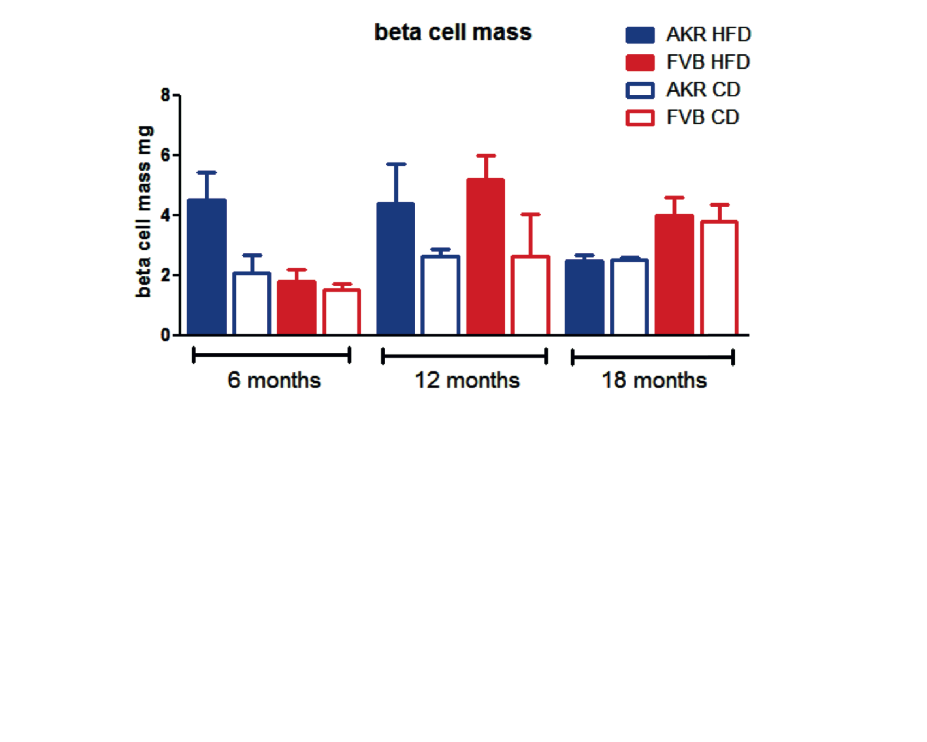


**Figure B.** **Increase in beta cell mass.** Beta cell mass for B6-mt^FVB^ strain (in red) and beta cell mass for B6-mt^AKR^ strain (in blue) under high-fat diet (filled boxes) and control diet (empty boxes).

We ignore any increase in the size of β-cells, such that the increase in β-cell mass constitutes only increase in β-cell number. We assume a logistic increase in β-cell proliferation.

$$\frac{d[BetaM]}{dt}=r_{b}\cdot\left(1-\frac{[BetaM]}{K_{b}}\right)\cdot[BetaM]$$

where, *r_b_* is the maximum growth rate of β-cell population and *K_b_* scales the maximum population size that the pancreatic islet can sustain.

**Sensitivity analysis**

Sensitivity analysis was performed by systematically varying the model parameters over a 100-fold range. Peak amplitude of the blood glucose levels and timing of the peak was plotted with respect change in parameter values (Figure C in S1 File).

**Figure C.** **Sensitivity analysis.** Parameters were varied systematically upto 10 fold higher (in red) and 10 fold lower (blue) than the default parameter value (green) (A). Amplitude of the blood glucose peak was plotted with respect to changing parameter value (B). Timing of the blood glucose peak was with respect to changing parameter value (C).

**Tables**

**Table A. Model parameters.**

| Parameter | Value | Source |
| --- | --- | --- |
| *k_1_* | 1.42 | [5] |
| *k_2_* | 15.6 | [5] |
| *k_3_* | 0.00272 | [5] |
| *k_4_* | 0.275 | Fitted |
| *k_5ml_* | 1.1 | Fitted |
| *k_5mi_* | 1.1 | Fitted |
| *k_6_* | 0.0018 | Fitted |
| *k_7l_* | 0.6 | Fitted |
| *k_7i_* | 0.4 | Fitted |
| *k_7m_* | 0.22 | Fitted |
| *σ_diet_* for CD | 18.72 | Fitted |
| *σ_diet_* for HFD | 18.72*1.4 | Fitted |
| *σ_fat_* | 9 | [20] |
| *k_ins_* | 0.778 | Fitted |
| *k_8_* | 0.195 | Fitted |
| *k_9_* | 0.02 | Fitted |
| *k_10_* | 0.213 | Fitted |
| *k_11_* for B6-mt^AKR^ | 0.132 | Fitted |
| *k_11_* for B6-mt^FVB^ | 0.132*0.1 | Fitted |
| *k_12_* | 0.0001 | Fitted |
| *k_13_* | 0.0011 | Fitted |
| *k_14_* | 0.066 | Fitted |
| *k_15_* | 0.045 | Fitted |
| *r_hf_* | 10 | Fitted |
| *r_cd_* | 0.1 | Fitted |
| a | 0.31 | Fitted |

**Table B. Parameters for β cell mass proliferation.**

| Parameter | Value |
| --- | --- |
| *r_b_* | CD=0.23,HFD=0.27 |
| *K_b_* | 6 |

**Table C. Parameters for high-fat diet induced low-grade inflammation.**

| Parameter | Value |
| --- | --- |
| *r_i_* | AKR=0.035, FVB=0.0375 |
| *K_i_* | AKR=22,FVB=18 |

**Table D. Initial conditions**

| Parameter | Value |
| --- | --- |
| G_0_ | 5 |
| I_0_ | 2 |
| L_0_ | 10 |
| FM_0_ | 1 |
| βM_0_ | 0.01 |
| Inflm_0_ | 0.01 |

The matlab code for the model is available at <http://www.sbi.uni-rostock.de/resources/software/glucose>.

# References

x

| 1. | Schwartz MW, Jr DP. Diabetes, obesity, and the brain. Science. 2005 Jan; 307(5708): p. 375-379. |
| --- | --- |
| 2. | Varela L, Horvath TL. Leptin and insulin pathways in POMC and AgRP neurons that modulate energy balance and glucose homeostasis. EMBO Rep. 2012 Dec; 13(12): p. 1079-1086. |
| 3. | Banks WA, Clever CM, Farrell CL. Partial saturation and regional variation in the blood-to-brain transport of leptin in normal weight mice. Am J Physiol Endocrinol Metab. 2000 Jun; 278(6): p. E1158--E1165. |
| 4. | Benoit SC, Clegg DJ, Seeley RJ, Woods SC. Insulin and leptin as adiposity signals. Recent Prog Horm Res. 2004; 59: p. 267-285. |
| 5. | Tam J, Fukumura D, Jain RK. A mathematical model of murine metabolic regulation by leptin: energy balance and defense of a stable body weight. Cell Metab. 2009 Jan; 9(1): p. 52-63. |
| 6. | van WH, Wyndham CH. An equation for prediction of energy expenditure of walking and running. J Appl Physiol. 1973 May; 34(5): p. 559-563. |
| 7. | Hoffman WP, Ness DK, L. RB. Analysis of rodent growth data in toxicology studies. Toxicol Sci. 2002 Apr; 66(2): p. 313-319. |
| 8. | Cumin F, Baum HP, Gasparo} M, Levens N. Removal of endogenous leptin from the circulation by the kidney. Int J Obes Relat Metab Disord. 1997 Jun; 21(6): p. 495-504. |
| 9. | Cammisotto PG, Bukowiecki LJ. Mechanisms of leptin secretion from white adipocytes. Am J Physiol Cell Physiol. 2002 Jul; 283(1): p. C244--C250. |
| 10. | Havel PJ. Role of adipose tissue in body-weight regulation: mechanisms regulating leptin production and energy balance. Proc Nutr Soc. 2000 Aug; 59(3): p. 359-371. |
| 11. | Ahrén B, Larsson H. Leptin--a regulator of islet function?: its plasma levels correlate with glucagon and insulin secretion in healthy women. Metabolism. 1997 Dec; 46(12): p. 1477-1481. |
| 12. | Malmström R, Taskinen MR, Karonen SL, Yki-Järvinen H. Insulin increases plasma leptin concentrations in normal subjects and patients with NIDDM. Diabetologia. 1996 Aug; 39(8): p. 993-996. |
| 13. | Sainsbury A, Cusin I, Doyle P, Rohner-Jeanrenaud F, Jeanrenaud B. Intracerebroventricular administration of neuropeptide Y to normal rats increases obese gene expression in white adipose tissue. Diabetologia. 1996 Mar; 39(3): p. 353-356. |
| 14. | Shimizu H, Ohtani K, Tsuchiya T, Takahashi H, Uehara Y, Sato N, et al. Leptin stimulates insulin secretion and synthesis in HIT-T 15 cells. Peptides. 1997; 18(8): p. 1263-1266. |
| 15. | Lumeng CN, Deyoung SM, Bodzin JL, Saltiel AR. Increased inflammatory properties of adipose tissue macrophages recruited during diet-induced obesity. Diabetes. 2007 Jan; 56(1): p. 16-23. |
| 16. | Weisberg SP, McCann D, Desai M, Rosenbaum M, Leibel RL, Ferrante, AW. Obesity is associated with macrophage accumulation in adipose tissue. J Clin Invest. 2003 Dec; 112(12): p. 1796-1808. |
| 17. | Jr AF. The immune cells in adipose tissue. Diabetes Obes Metab. 2013 Sep; 15 Suppl 3: p. 34-38. |
| 18. | Xu H, Barnes GT, Yang Q, Tan G, Yang D, Chou CJ, et al. Chronic inflammation in fat plays a crucial role in the development of obesity-related insulin resistance. J Clin Invest. 2003 Dec; 112(12): p. 1821-1830. |
| 19. | Gerlee P. The model muddle: in search of tumor growth laws. Cancer Res. 2013 Apr; 73(8): p. 2407-2411. |
| 20. | Schraer WD, Stoltze HJ. Biology: Study of Life. Seventh Edition ed.: Pearson Prentice Hall; 1999. |

x
